# Safer and Sustainable-by-Design Hydroxyapatite Nanobiomaterials for Biomedical Applications: Assessment of Environmental Hazards

**DOI:** 10.3390/nano12224060

**Published:** 2022-11-18

**Authors:** Susana I. L. Gomes, Bruno Guimarães, Elisabetta Campodoni, Monica Sandri, Simone Sprio, Magda Blosi, Anna L. Costa, Janeck J. Scott-Fordsmand, Mónica J. B. Amorim

**Affiliations:** 1 Department of Biology & CESAM, University of Aveiro, 3810-193 Aveiro, Portugal; 2 National Research Council, Institute of Science and Technology for Ceramics, 48018 Faenza, RA, Italy; 3 Department of Ecoscience, Aarhus University, C.F. Møllers Alle 4, DK-8000 Aarhus, Denmark

**Keywords:** biomaterials, health care applications, hydroxyapatite nanomaterials, toxicity, enchytraeids, soil

## Abstract

Developments in the nanotechnology area occur ensuring compliance with regulatory requirements, not only in terms of safety requirements, but also to meet sustainability goals. Hence, safer and sustainable-by-design (SSbD) materials are also aimed for during developmental process. Similar to with any new materials their safety must be assessed. Nanobiomaterials can offer large advantages in the biomedical field, in areas such as tissue repair and regeneration, cancer therapy, etc. For example, although hydroxyapatite-based nanomaterials (nHA) are among the most studied biomaterials, its ecotoxicological effects are mostly unknown. In the present study we investigated the toxicity of seven nHA-based materials, covering both different biomedical applications, e.g., iron-doped hydroxyapatite designed for theragnostic applications), hybrid collagen/hydroxyapatite composites, designed for bone tissue regeneration, and SSbD alternative materials such as titanium-doped hydroxyapatite/alginate composite, designed as sunscreen. The effects were assessed using the soil model *Enchytraeus crypticus* (Oligochaeta) in the natural standard LUFA 2.2 soil. The assessed endpoints included the 2, 3 and 4 days avoidance behavior (short-term), 28 days survival, size and reproduction (long term based on the OECD standard reproduction test), and 56 days survival and reproduction (longer-term OECD extension). Although overall results showed little to no toxicity among the tested nHA, there was a significant decrease in animals’ size for Ti-containing nHA. Moreover, there was a tendency for higher toxicity at the lowest concentrations (i.e., 100 mg/kg). This requires further investigation to ensure safety.

## 1. Introduction

Nanotechnology is a multi-million-dollar industry, estimated to reach a market size of approx. 34 billion dollars by 2030. The diversity of applications ranges from energy and electronics to healthcare, cosmetics, aerospace, etc. [1]. As outlined in the United Nations Sustainable Development Goals 2030, any new material or product should not only be functional and cost-effective but also safe and sustainable. This is to ensure compliance with regulation and promote a clean environment, aligned with a zero-pollution ambition [2]). The implementation of safe and sustainable by design (SSbD) materials [3,4] offers nanotechnology a large advantage, due to the cost of developing and testing materials. Safe and sustainable by design refers to the process of anticipating potential toxic impacts of a product or material on human and/or environmental health, and to address these concerns during the innovation process, i.e., altering the product design if needed before marketing [5].

Nanobiomaterials—natural or synthetic nanoscale materials, that interact with biological systems to perform a specific function, e.g., treat, support, or replace damaged tissue—have a tremendous potential in biomedical applications, e.g., tissue repair and regeneration, drug and gene delivery, cancer therapy, medical imaging, and theragnostics [6]. Hydroxyapatite (HA) is a calcium phosphate-based compound which is among the most studied biomaterials in the medical field due to its biocompatibility and for being the main constituent of the mineral part of bone and teeth [7,8]. The low mechanical properties of HA ceramic are surpassed by its combination with a large number of polymers (e.g., collagen, polylactic acid, alginates, polyethylene) producing nanocomposites with broader applications [8], e.g., photothermal therapy [9]. Despite the great promise of HA based nanomaterials (nHA) in the biomedical field, its ecotoxicity must still be thoroughly assessed to ensure that it is safe and sustainable.

The available data on nHA are often inconsistent (please note that in the vast majority of the studies, the tested nHA were different nHA forms which hampers the comparison). Some in vitro studies report induction of inflammation, but no cytotoxicity to various cell lines [10,11,12]. However, the induction of cyto-toxicity (cell death) following nHA exposure has also been reported [13,14,15]. Cytotoxicity correlated with nHA uptake and cell load, suggesting that the increase in cytoplasmic calcium levels, as result of intracellular dissolution of nHA over time, is likely the cause of cell death [13]. Nevertheless, Zhao et al. [14] found that shape has a more important role on nHA cytotoxicity than cell uptake, as needle- and plate-shaped nHA were more toxic to BEAS-2B cells than rod-shaped nHA, the latter for which higher particle–cell association and increased cellular uptake were observed [14]. Size has been shown to influence the cytotoxicity of sphere-shaped nHA to HepG2 cell [15], with higher toxicity of the 45 nm nHA (in comparison to 26, 78 and 175 nm nHA). The higher toxicity was associated with higher levels of apoptosis markers and higher concentration of nHA around the nucleus of the cells [15]. Among the in vivo studies available, rod-shaped nHA caused no effects to wistar rats exposed through food for 1 year (evaluated parameters were body weight, food consumption, clinical observations, survival, biochemical and hematological analysis, and histopathological evaluation) [16]. A study on *Drosophila melanogaster* showed no evidence of toxicity in terms of biochemical parameters (lipid peroxidation and nitro blue tetrazolium assay), behavioral changes in larvae and adults, nor chronic survival toxicity [17]; the nHA used was 20 nm with the presence of needle-like structures. On the other hand, rod-shaped nHA with 70–80 by 40–50 nm induced oxidative stress and cell damage to gut cell of *D. melanogaster* larvae, caused developmental delay in the late third instar larvae, decreased hatching, and behavior and phenotypical abnormalities [18]. The effects reported were attributed to interference of nHA with the calcium and phosphorus absorption pathway [18]. Few studies have analyzed in vitro and in vivo effects simultaneously. Needle- and rod-shaped nHA caused no cytotoxicity (in vitro) to catfish B-cells (3B11) and catfish T-cells (28s.3) up to 300 μg/mL; however, hatching inhibition was reported in zebrafish (in vivo) and, although all the larvae hatched at 120 h post fertilization, needle-shaped nHA caused spine malformation in 75% of the embryos [19]. In another study, nHA caused no cytotoxicity to murine fibroblast cells (L929), but induced an increase in lipid peroxidation levels in rat liver cells (in vitro) [20]. The same study reported no effects to guinea pigs, in vivo, when evaluating skin sensitization potential, blood parameters, oxidative stress in liver and brain and DNA damage [20]. It is noticeable that ecotoxicity information on nHA is missing, representing a major current gap for safe and sustainable materials.

The aim of the present study was to investigate the ecotoxicological effects of seven HA or HA-hybrid nanomaterials. These HA materials [part of the repository of test materials within the EU H2020-NMBP-2017 BIORIMA project (GA 760928)] covered various biomedical applications and SSbD alternative materials, i.e., (1) commercial hydroxyapatite was purchased from Sigma-Aldrich (Merck Life Science S.L.U., Portugal) (Sigma-HA)—used as a standard for comparison, (2) calcium hydroxyapatite (CaP-HA), (3) superparamagnetic iron doped hydroxyapatite (Fe-HA) [21]—a bioresorbable superparamagnetic nanoparticle proposed for theragnostic applications, (4) titanium-doped hydroxyapatite (Ti-HA), and (5) hybrid alginate/titanium-doped hydroxyapatite (Ti-HA-Alg) [22,23]—proposed an alternative to TiO_2_ NPs to boost SPF in sunscreens, as they display high biocompatibility, bands of absorption/reflection of the UV radiation in the range suitable for application as sunscreen, and no photocatalytic effect thanks to the absence of titanium oxide crystals, and (6) collagen/hydroxyapatite hybrid scaffold (HA-Coll) [24], and (7) collagen/iron-doped hydroxyapatite hybrid scaffold (Fe-HA-Coll) [25]—proposed as bio-hybrid materials for regenerative medicine, as scaffolds are biocompatible and display good ability to stimulate and support cell adhesion and proliferation, holding promise, for instance, for therapies targeting bone tissue regeneration [26]. For the ecotoxicology study, the globally important soil invertebrate model species *Enchytraeus crypticus* (Oligochaeta) [27] was used. The effects were investigated based on the OECD standard 28 days enchytraeid reproduction test (ERT) [27] and on a longer-term exposure of 56 days, a standard extension. Further endpoints were included, i.e., animal size and intermediate sample times (7, 14, 21 days). In addition, for 2 of the nHA materials (6 and 7) it was not possible to homogeneously mix these with the soil, hence a short-term avoidance behavior test was used as screening tool to see if the organisms could detect the materials. In addition, the standard 2 days test was enhanced with additional sample times, i.e., 2, 3 and 4 days.

## 2. Materials and Methods

### 2.1. Test Organism

The test species *Enchytraeus crypticus*, (Westheide and Graefe, 1992) was used. The cultures were kept in agar, consisting of Bacti-Agar medium (Oxoid, Agar No. 1) and a sterilized mixture of four different salt solutions at final concentrations of 2 mM CaCl_2_·2H_2_O, 1 mM MgSO_4_, 0.08 mM KCl, and 0.75 mM NaHCO_3_, under controlled conditions of temperature (19 ± 1 °C) and photoperiod (16:8 h light:dark). The animals were fed on ground autoclaved oats twice per week.

### 2.2. Test Soil

LUFA 2.2 natural standard soil (LUFA Speyer, Germany) was used. Its main characteristics are pH (0.01 M CaCl_2_): 5.6 ± 0.4; organic carbon: 1.71 ± 0.30%; cation exchange capacity (CEC): 9.2 ± 1.4 meq/100 g; maximum water holding capacity (maxWHC): 44.8 ± 2.9 g/100 g; and grain size distribution of: 8.0 ± 1.5% clay, 13.7 ± 1.0% silt, and 78.3 ± 1.0% sand content.

### 2.3. Test Materials, Synthesis, Characterization, and Spiking

The test materials included: (1) the commercial hydroxyapatite (HA) purchased from Sigma-Aldrich (Merck Life Science S.L.U., Portugal) [CAS No. 12167-74-7, nanopowder, <200 nm particle size (BET), ≥97%, synthetic] (further referred to as Sigma-HA), used as commercial standard for comparison, (2) calcium hydroxyapatite (further referred to as CaP-HA) as standard of lower crystallinity, (3) superparamagnetic iron doped hydroxyapatite nanoparticles (further referred to as Fe-HA), (4) titanium-doped hydroxyapatite nanoparticles (further referred to as Ti-HA), (5) alginate/titanium-doped hydroxyapatite hybrid particles (further referred to as Ti-HA-Alg), (6) collagen/hydroxyapatite hybrid scaffold (further referred to as HA-Coll), and (7) collagen/iron-doped hydroxyapatite hybrid scaffold (further referred to as Fe-HA-Coll).

All of the synthesized materials were made available within the BIORIMA project materials repository. Briefly, CaP-HA, Fe-HA, Ti-HA were prepared by aqueous precipitation reaction. For CaP-HA, ortho-phosphoric acid solution (H_3_PO_4_, 85 wt.%; Sigma Aldrich, Saint Louis, MO, USA) was dropped onto calcium hydroxide solution (Ca(OH)_2_, 95 wt.%; Sigma Aldrich, Saint Louis, MO, USA), at 40 °C under vigorous stirring; stirring and temperature were kept for 2 h after the addition finished. For Fe-HA, ortho-phosphoric acid solution was dropped onto calcium hydroxide containing Fe^2+^/^3+^ solution (FeCl_2_·4H_2_O, FeCl_3_·6H_2_O; Sigma Aldrich, Saint Louis, MO, USA), at 45 °C under vigorous stirring; stirring and temperature were kept for 3 h after the addition finished. For Ti-HA, ortho-phosphoric acid solution and titanium isopropoxide (Ti(iOPr)_4_, 97 wt.%; Sigma Aldrich, Saint Louis, MO, USA) in isopropanol (C3H8O, Sigma Aldrich, Saint Louis, MO, USA) solution were simultaneously dropped onto calcium hydroxide solution, at 45 °C under vigorous stirring; stirring and temperature were kept for 3 h after the addition finished. For CaP-HA, Fe-HA and Ti-HA, after stirring the reactions were let without stirring or heat overnight. After that, the particles were resuspended by stirring for 15 min, the pellet was collected by centrifugation, and washed with water 3 times. The final powders were dried at 40 °C and sieved under 150 μm.

The hybrid materials Ti-HA-Alg, HA-Coll and Fe-HA-Coll were produced by a nature-inspired biomineralization process, consisting of the heterogeneous nucleation of titanium/iron-doped hydroxyapatite NPs (Ti/Fe-HA) on biodegradable natural polymers: alginate (alginic acid sodium salt from brown algae; Sigma Aldrich, Saint Louis, MO, USA) or collagen (Opocrin SPA, Modena, Italy). In both cases, ortho-phosphoric acid solution added with the polymer (Alg or Coll), was dropped onto calcium hydroxide containing Ti or Fe^2+^/^3+^ solution, at room temperature under gentle stirring; maturation at room temperature was kept for 1 h after the addition finished. Subsequently, the hybrid product was washed with water 3 times by sieving the gel and finally it was dried by freeze-drying process to obtain porous structures/scaffolds. For Ti-HA-Alg, to achieve microparticles, the dried product was sieved under 150 μm, then micronized in dry and cold conditions.

Nanomaterials were characterized by Transmission Electron Microscopy (TEM; Tecnai F20, FEI, Hillsboro, OR, USA), Scanning Electron Microscopy (SEM; Environmental Scanning Electron Microscope, Quanta 600 FEG, FEI Company, Hillsboro, OR, USA), Field Emission-Scanning Electron Microscopy (FE-SEM; Field Emission Gun Scanning Electron Microscope, FEI, Quanta 200, USA), X-ray Diffraction spectroscopy (XRD; D8 Advance diffractometer model, Bruker, Karlsruhe, Germany), Dynamic Light Scattering (DLS), Zeta-Potential (dynamic light scattering with a Zetasizer Nano ZS, Malvern Ltd., UK), Thermo Gravimetric Analysis (TGA; Simultaneous Thermal Analyzer, STA 409C, Netsch, Germany), and UV-Vis spectroscopy (UV-Vis-NIR spectrophotometer Lambda 750; Perkin Elmer Instrument, USA). For full details please see Supplementary Materials.

All the materials were added to soil as dry powders as recommended for non-soluble materials [28]. Each replicate was spiked individually to ensure total raw amounts of the tested material. The soil was homogeneously mixed, deionized water was added until 50% of soil’s maxWHC, and the soil was mixed again. The soil was left to equilibrate for 1 day prior the test start. The concentration ranges and tests performed for each material is presented on Table 1.

HA-Coll and Fe-HA-Coll have a fibrous structure and when mixed onto soil a homogeneous distribution was not possible. The exposure to spiked media that is not homogeneously distributed will not allow for the assessment of effects as a consequence of exposure, and high variability is expected. Hence, avoidance tests were performed (instead of the reproduction tests) to assess enchytraeids behavior.

### 2.4. Test Procedures

#### 2.4.1. Reproduction Tests

The tests followed the standard guideline for the Enchytraeid Reproduction Test (ERT, 28 days) [27], plus the OECD extension, as described in, e.g., Ribeiro et al. [29]. In short, the test was extended for 28 additional days (56 days in total) and extra monitoring sampling times at days 7, 14, 21, (28) and 56. Endpoints included survival for all sampling periods, reproduction at days 28 and 56, i.e., number of juveniles and population, respectively, and size at day 28. Four replicates per treatment were carried out, except at days 7, 14 and 21 (1 replicate). At test start, ten synchronized age organisms (young, 18–20 days old after cocoon laying) were introduced in each test vessel with moist soil (7, 14, 21, and 28-days exposure: Ø4 cm vessel, 20 g of soil, and 56 days exposure: Ø5.5 cm vessel, 40 g of soil) and food supply (22 ± 2 mg, autoclaved rolled oats). The test ran up to 56 days at 20 ± 1 °C and 16:8 h photoperiod. Food (11 ± 1 mg: until day 28, and 33 ± 3 mg: from 28 to 56 days) and water (based on weight loss) was replenished weekly. On sampling days 7, 14, 21, and 28, adults were carefully removed from the soil and counted (survival). The juveniles were counted at day 28 and 56 using a stereo microscope, to assess reproduction. After being fixed for 24 h with ethanol and Bengal rose (Merck Life Science S.L.U., Portugal) (1% in ethanol), soil samples were sieved through meshes with decreasing pore size (1.6, 0.5, and 0.3 mm) in order to separate the enchytraeids from most of the soil and facilitate counting. For the replicates that continued until day 56, adults were carefully removed from the soil at day 28. The adult organisms collected at day 28 were photographed after staining with Bengal rose, and size (area, mm2) was assessed using the software ImageJ (v.1.52a, Wayne Rasband, National Institutes of Health, USA) [30].

#### 2.4.2. Avoidance Tests

Avoidance tests were performed following the earthworm avoidance test guideline [31] using *E. crypticus* with adaptations as described in Bicho et al. [32]. In short, plastic containers (2.5 × 6.5ø cm) with one removable plastic divider were used; each replicate contained 50 g of soil, 25 g in each side, i.e., the control and spiked soil. After this, the wall was gently removed and ten adult organisms with well-developed clitellum were placed on the contact line of the soils. The boxes were covered with a lid (containing small holes) and kept, for 48, 72 and 96 h, at 20 ± 1 °C and a photoperiod of 16:8 h (light–dark). Five replicates per treatment were used (at 48 h and 72 h monitoring, the replicates were checked but kept until the 96 h sample). At the end of the test period, the divider was again inserted in the separation line between the two soils and each side of the box was independently searched for worms.

### 2.5. Data Analysis

For all the tests, to assess differences between treatments and controls, one-way analysis of variance (ANOVA) was performed, followed by the post hoc Dunnett’s method for multiple comparisons at a significance level of 0.05 (SigmaPlot v.14.0, Systat Software, Inc., San Jose, CA, USA). Avoidance was calculated as the percentage of worms that avoided the treated soil in the test container from the total number of worms in that container. The mean percentages of net responses (NR) were calculated as follows (Equation (1)):

(1) $$NR=\frac{C-T}{N}\times 100,$$

where *C* is the number of organisms observed in the control soil, *T* is the number of organisms observed in test soil and *N* is the total number of organisms per replicate. A positive (+) *NR* indicates avoidance, and a negative (−) *NR* indicates a non-response (or attraction) to the test substance

## 3. Results

### 3.1. Materials Characterization

A summary of the main characteristics of the nHAs tested is provided in Table 2. For further details please see SI. In summary, among the “powder”, Sigma-HA forms the least stable suspension (larger aggregates—larger hydrodynamic diameter and surface charge closer to zero); CaP-HA, Fe-HA and Ti-HA forms smaller aggregates and are negatively charged. The substitution of HA atoms by Fe or Ti changes the shape of the nHA from round to needle- or rod-like, for Fe-HA and Ti-HA, respectively, also affected by the temperature condition during synthesis and maturation step. The agglomerated state of Ti-HA-Alg hybrid particles and the fibrous structure of HA-Coll and Fe-HA-Coll are clearly shown in SEM images (Figures S1–S7).

### 3.2. Ecotoxicological Tests

The validity criteria were fulfilled as within the standard OECD test, i.e., in controls, adult mortality < 20% and the number of juveniles > 50 per replicate, with a coefficient of variation < 50%.

**Figure 1:**
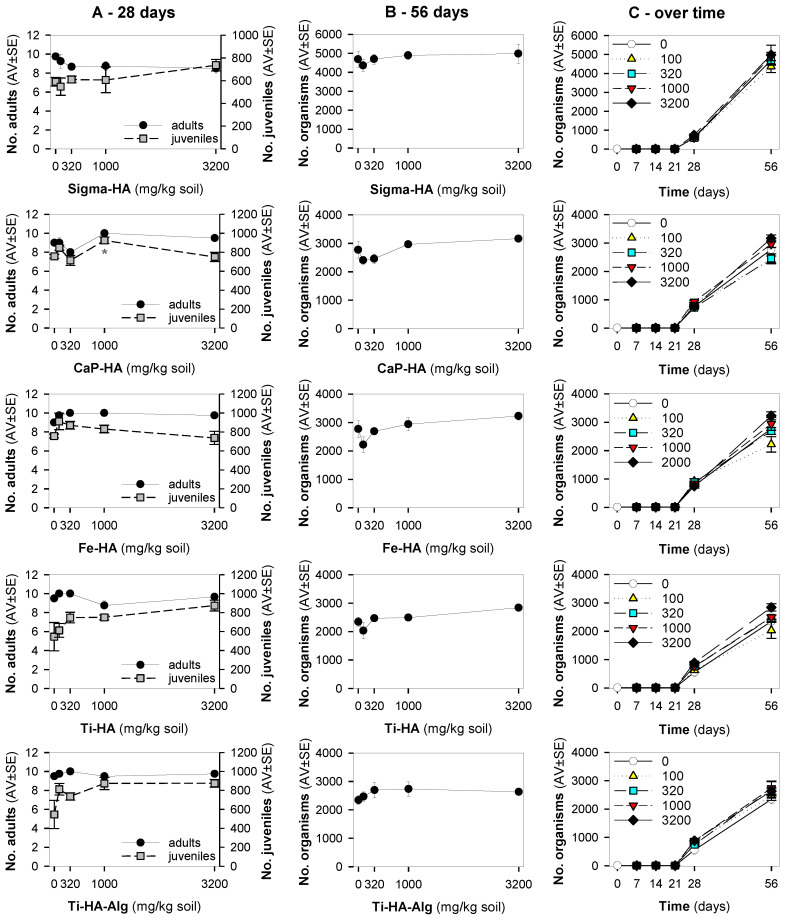
The results in terms of survival and reproduction from the Enchytraeid Reproduction Test, when exposing *Enchytraeus crypticus* in LUFA 2.2 soil to the commercial hydroxyapatite (Sigma-HA), calcium hydroxyapatite (CaP-HA), superparamagnetic iron doped hydroxyapatite (Fe-HA), and titanium-doped hydroxyapatite nanoparticles (Ti-HA), alginate/titanium-doped hydroxyapatite hybrid particles (Ti-HA-Alg) during (**A**) 28 days (OECD Standard), (**B**) 56 days (OECD standard extension), and (**C**) overview of the time series sampling at days: 7, 14, 21, 28 and 56 days. Values represent number of adults, juveniles, and population as average ± standard error (AV ± SE). *: *p* < 0.05 (Dunnett’s method).

**Figure 2:**
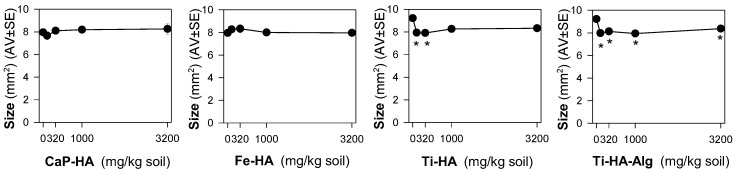
The results in terms of adults’ size from the Enchytraeid Reproduction Test, when exposing *Enchytraeus crypticus* in LUFA 2.2 soil to calcium hydroxyapatite (CaP-HA), superparamagnetic iron doped hydroxyapatite (Fe-HA), and titanium-doped hydroxyapatite nanoparticles (Ti-HA), and alginate/titanium-doped hydroxyapatite hybrid composite (Ti-HA-Alg), for 28 days. Values represent adults’ size (area, mm^2^) as average ± standard error (AV ± SE). *: *p* < 0.05 (Dunnett’s method).

The results showed that in the standard exposure test (28 days) no dose-response negative impact pattern occurred in terms of survival or reproduction (Figure 1A). For Ti-HA and TiHA-Alg, while there was an increase in reproduction, the size of the animals was reduced (Figure 2). For the standard extension exposure (56 days), although there was no dose-response pattern, there was a decrease in population numbers observed at the lowest tested concentration (100 mg NM/kg soil) for all materials, except Ti-HA-Alg (Figure 1B,C).

**Figure 3:**
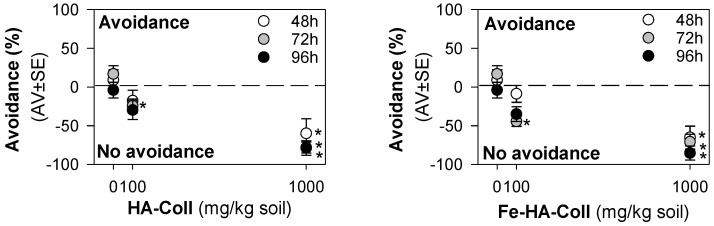
The results in terms of avoidance behavior from the avoidance test when exposing *Enchytraeus crypticus* in LUFA 2.2 soil, during 48 (standard), 72, and 96 h to collagen/hydroxyapatite hybrid scaffold (HA-Coll) and collagen/iron-doped hydroxyapatite hybrid scaffold (Fe-HA-Coll). Values represent average ± standard error (AV ± SE). *: *p* < 0.05 (Dunnett’s method).

For the avoidance test, the validity criteria were fulfilled, i.e., less than 20% mortality and homogeneous distribution (no avoidance) in controls. The results showed that enchytraeids did not avoid the soil containing HA-Coll or Fe-HA-Coll, instead there was a dose-dependent preference for the spiked soil (Figure 3). The same pattern was observed at all exposure periods (48, 72, 96 h).

## 4. Discussion

No dose-response impact of the nHA-based materials was observed in terms of survival and reproduction in the standard test (28 days), but in the extended exposure (56 days) the lowest concentration caused an impact to *E. crypticus*. Higher effects at lower or intermediate concentrations of NMs have been previously reported to soil invertebrates exposed to, e.g., Ag [33,34], Ni [35], and Au [36]. The source of this increased toxicity is probably related to higher dispersibility/lower agglomeration of the particles at lower concentrations, which results in a higher availability. This was also reported for nHA in aqueous media, by Zhao et al. [19] whose results showed highest effect in terms of zebrafish hatching and embryo development at the lowest tested concentration (3 μg/mL), also associated with the lowest particles agglomeration.

Furthermore, it was of interest to note that there was a combination between a significant reduction in the size of animals exposed to Ti-HA and Ti-HA-Alg for 28 days, and their increased number of juveniles. This is not an uncommon situation, where stressed animals respond by increasing one on trait compromising another, i.e., increase the reproduction compromising growth, hence prioritizing energy allocation to the next generation numbers. The growth inhibition can result from impairment in feeding activity/damage to digestive track [37], or could be related to developmental effects. In *D. melanogaster*, rod-shaped nHA exposure, caused developmental delay in the late third instar larvae, among other effects including phenotypical abnormalities in the hatched flies [18]. Considering that size was only reduced for Ti-doped NMs, Ti could be the source of the observed effects. TiO_2_ NPs inhibit growth in zebrafish [38]. It is known that Ti can be released from Ti-based bone implants applied in mini pig maxillae [39], thus Ti release from the NMs and consequent toxicity cannot be ruled out. The extended exposure of 56 days showed that Ti-HA (100 mg/kg) caused a reduction in the population numbers, but this was not the case for Ti-HA-Alg.

Enchytraeids showed a clear attraction behavior or preference for HA-Coll and Fe-HA-Coll spiked soil, independently of the exposure period. Previous studies have shown that the attraction for spiked soil can be caused by neurotoxic effects resulting in the animals’ incapacity to avoid the spiked soil [32,40]. However, it was observed that animals feed on Fe-HA-Coll; because of its orange color, Fe-HA-Coll was easily visualized inside the animals’ gut (Figure S8). HA-Coll must also be ingested, although being whitish it could not be easily identified. This could be somehow associated to the preference for the spiked soil, a palatable material. Neurotoxicity seems unlikely to be the reason considering the little to no effects observed in terms of survival and reproduction, for the majority of the nHA, and including the Fe-HA material.

Although the role of shape or size on nHA toxicity has been highlighted [14,15,19], our results do not allow for the confirmation of any association. For example, Ti-HA, which caused a decrease in animals’ growth, has a rod-shape, whereas Sigma-HA and CaP-HA are spherical and Fe-HA look more needle-like (Figure S3). Since the tested NMs have other aspects varying besides shape or size, any assumption would be more of a speculation.

## 5. Conclusions

We provided ecotoxicological data for seven nHA materials, which was a current gap in the literature. Considering the overall low ecotoxicity to *E. crypticus*, the nHA tested materials do not seem to cause high risk to soil inhabiting invertebrates. Special attention should be given to lower concentrations of NMs, for nHA materials in the 100–320 mg/kg range, where increased impacts were observed. From all nHA SSbD material options, since Ti-HA and Ti-HA-Alg caused growth inhibition, likely due to the presence of Ti—these nHA seemed to be the least safe.

## Figures and Tables

**Table 1:** The summary of the test type, endpoints, and tested concentrations for each test material: commercial hydroxyapatite (Sigma-HA), calcium hydroxyapatite (CaP-HA), superparamagnetic iron doped hydroxyapatite (Fe-HA), titanium-doped hydroxyapatite nanoparticles (Ti-HA), alginate/titanium-doped hydroxyapatite hybrid particles (Ti-HA-Alg), collagen/hydroxyapatite hybrid scaffold (HA-Coll), and collagen/iron-doped hydroxyapatite hybrid scaffold (Fe-HA-Coll).

Test Material	Test Type	Endpoints: Sampling Days	Concentrations (mg NM/kg Soil)	Visual Aspect
Sigma-HA	ERT ERT extension	Survival: 7, 14, 21, 28; Reproduction: 28, 56	0, 100, 320, 1000, 3200	Powder
CaP-HA	ERT ERT extension	Survival: 7, 14, 21, 28; Reproduction: 28, 56; Size: 28	0, 100, 320, 1000, 3200	Powder
Fe-HA	ERT ERT extension	Survival: 7, 14, 21, 28; Reproduction: 28, 56; Size: 28	0, 100, 320, 1000, 3200	Powder
Ti-HA	ERT ERT extension	Survival: 7, 14, 21, 28; Reproduction: 28, 56; Size: 28	0, 100, 320, 1000, 3200	Powder
Ti-HA-Alg	ERT ERT extension	Survival: 7, 14, 21, 28; Reproduction: 28, 56; Size: 28	0, 100, 320, 1000, 3200	Flakes
HA-Coll	Avoidance	Avoidance behaviour: 2, 3, 4	0, 100, 1000	Aggregated Fibbers
Fe-HA-Coll	Avoidance	Avoidance behaviour: 2, 3, 4	0, 100, 1000	Aggregated Fibbers

**Table 2:** A summary of the tested nHA (commercial hydroxyapatite (Sigma-HA), calcium hydroxyapatite (CaP-HA), superparamagnetic iron doped hydroxyapatite (Fe-HA), and titanium-doped hydroxyapatite nanoparticles (Ti-HA), alginate/titanium-doped hydroxyapatite hybrid particles (Ti-HA-Alg), collagen/hydroxyapatite hybrid scaffold (HA-Coll) and collagen/iron-doped hydroxyapatite hybrid scaffold (Fe-HA-Coll)) main characteristics, including chemical composition, hydrodynamic size, surface charge and shape based on TEM/SEM pictures. DLS: dynamic light scattering, TEM: transmission electron microscopy, SEM: scanning electron microscopy.

	nHA						
	Sigma-HA	CaP-HA	Fe-HA	Ti-HA	Ti-HA-Alg	HA-Coll	Fe-HA-Coll
Chemical composition	Ca_5_(OH) (PO_4_)_3_	Ca = 34 wt%; P = 16 wt%; Ca/P = 1.64 mol/mol	Ca = 23 wt%; P = 15 wt%; Fe = 10 wt%; (Ca + Fe)/P = 1.64 mol/mol	Ca = 37.0 wt%; P = 14.6 wt%; Ti = 4.5 wt%; (Ca + P)/(P + Ti) = 1.49 mol/mol	Ca/P = 1.60 mol; Ti/Ca = 15 mol%; Ti/P = 23.2 mol%; (Ca + Ti)/(P + Ti)= 1.49 mol/mol Alg:TiHA = 10:90	Ca/P = 1.60 mol/mol HA:Coll = 60:40	Ca/P = 1.43 mol/mol; Fe/Ca = 0.19 mol%; (Ca + Fe)/P = 1.70 mol/mol; Fe = 3.83 wt% HA:Coll = 60:40
Hydrodynamic size (DLS, nm)	2441	319.36	163.7	241.7	-	-	-
Surface charge (zeta potential, mV)	1.35	−19.66	−19.3	−9.11	-	-	-
Shape (TEM/SEM)	Round	Round	Needle-like	Rod-like	Hybrid flakes	Hybrid fibres	Hybrid fibres
	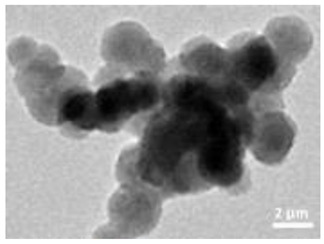	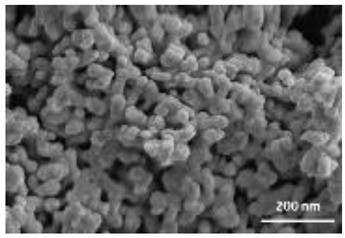	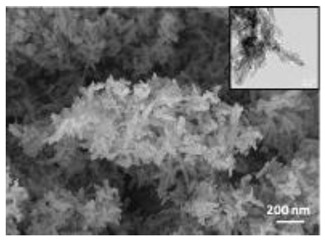	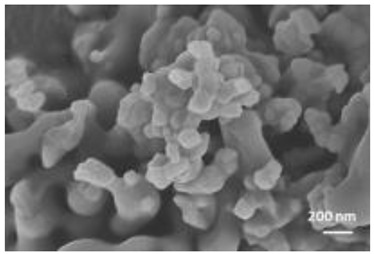	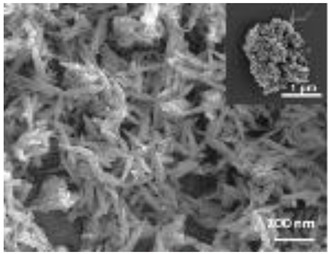	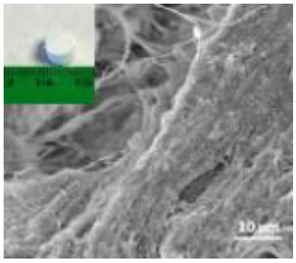	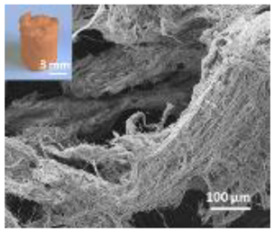

## Data Availability

The data presented in this study are available on request from the corresponding author.

## Supplementary Materials

The following supporting information can be downloaded at: https://www.mdpi.com/article/10.3390/nano12224060/s1, Figure S1: Images of Sigma-HA via Transmission Electron Microscopy (TEM): (A,B) and via Scanning Electron Microscopy (SEM): (C,D); Figure S2: CaP-HA NM characterization via (A) SEM-FEG image, (B) XRD spectrum and (C) FTIR spectrum; Figure S3: Fe-HA NM characterization via (A) SEM-FEG image, inset) TEM image, (B) XRD spectrum, and (C) FTIR spectrum; Figure S4: Ti-HA NM characterization via (A) FEG-SEM image, (B) XRD spectrum, and (C) FTIR spectrum; Figure S5: Ti-HA-Alg NM characterization via (A) FEG-SEM image, (B) TGA spectrum, (C) XRD spectrum, and (D) FTIR spectrum; Figure S6: HA-Coll hybrid composite characterization via (A) ESEM image, (B) TGA spectrum, (C) XRD spectrum and (D) FTIR spectrum; Figure S7: Fe-HA-Coll hybrid composite characterization via (A) ESEM image, (B) TGA spectrum, (C) XRD spectrum and (D) FTIR spectrum; Figure S8: Images from the avoidance test at the end of the exposure (4 days) with Fe-HA-Coll (1000 mg/kg soil): (A) detail of Fe-HA-Coll agglomerate—red arrow—visible in soil; (B) *Enchytraeus crypticus* as sampled and placed in water at the end of the test, showing the presence of Fe-HA-Coll in the gut—red arrows. Refs. [22,23,25,41,42,43] are cited in the Supplementary Materials.

## References

[1] Nanotechnology Market by Type (Nanosensor and Nanodevice) and Application (Electronics, Energy, Chemical Manufacturing, Aerospace & Defense, Healthcare, and Others): Global Opportunity Analysis and Industry Forecast, 2021–2030. https://www.alliedmarketresearch.com/nanotechnology-market.

[2] European Commission (2021). Pathway to a Healthy Planet for All—EU Action Plan: “Towards Zero Pollution for Air, Water and Soil”. https://lifeproetv.eu/wp-content/uploads/2022/02/8P_J-DEugenio_EU_policy_framework_Zero_Pollution_Action_Plan_and_ETV.pdf.

[3] Gottardo S., Mech A., Drbohlavová J., Małyska A., Bøwadt S., Riego Sintes J., Rauscher H. (2021). Towards safe and sustainable innovation in nanotechnology: State-of-play for smart nanomaterials. NanoImpact.

[4] Salieri B., Barruetabeña L., Rodríguez-Llopis I., Jacobsen N.R., Manier N., Trouiller B., Chapon V., Hadrup N., Jiménez A.S., Micheletti C. (2021). Integrative approach in a safe by design context combining risk, life cycle and socio-economic assessment for safer and sustainable nanomaterials. NanoImpact.

[5] Hjorth R., van Hove L., Wickson F. (2017). What can nanosafety learn from drug development? The feasibility of “safety by design”. Nanotoxicology.

[6] Ramburrun P., Khan R.A., Choonara Y.E. (2022). Design, preparation, and functionalization of nanobiomaterials for enhanced efficacy in current and future biomedical applications. Nanotechnol. Rev..

[7] Pepla E. (2014). Nano-hydroxyapatite and its applications in preventive, restorative and regenerative dentistry: A review of literature. Ann. Stomatol..

[8] Andronescu E., Grumezescu A.M., Guşă M.-I., Holban A.M., Ilie F.-C., Irimia A., Nicoară I.-F., Ţone M. (2016). Nano-hydroxyapatite. Nanobiomaterials in Hard Tissue Engineering.

[9] Wang Y.-C., Dai H.-L., Li Z.-H., Meng Z.-Y., Xiao Y., Zhao Z. (2021). Mesoporous polydopamine-coated hydroxyapatite nano-composites for ROS-triggered nitric oxide-enhanced photothermal therapy of osteosarcoma. J. Mater. Chem. B.

[10] Huang J., Best S.M., Bonfield W., Brooks R.A., Rushton N., Jayasinghe S.N., Edirisinghe M.J. (2004). In vitro assessment of the biological response to nano-sized hydroxyapatite. J. Mater. Sci. Mater. Med..

[11] Zhao X., Heng B.C., Xiong S., Guo J., Tan T.T.-Y., Boey F.Y.C., Ng K.W., Loo J.S.C. (2011). In vitro assessment of cellular responses to rod-shaped hydroxyapatite nanoparticles of varying lengths and surface areas. Nanotoxicology.

[12] Albrecht C., Scherbart A.M., van Berlo D., Braunbarth C.M., Schins R.P.F., Scheel J. (2009). Evaluation of cytotoxic effects and oxidative stress with hydroxyapatite dispersions of different physicochemical properties in rat NR8383 cells and primary macrophages. Toxicol. In Vitr..

[13] Motskin M., Wright D.M., Muller K., Kyle N., Gard T.G., Porter A.E., Skepper J.N. (2009). Hydroxyapatite nano and microparticles: Correlation of particle properties with cytotoxicity and biostability. Biomaterials.

[14] Zhao X., Ng S., Heng B.C., Guo J., Ma L., Tan T.T.Y., Ng K.W., Loo S.C.J. (2013). Cytotoxicity of hydroxyapatite nanoparticles is shape and cell dependent. Arch. Toxicol..

[15] Yuan Y., Liu C., Qian J., Wang J., Zhang Y. (2010). Size-mediated cytotoxicity and apoptosis of hydroxyapatite nanoparticles in human hepatoma HepG2 cells. Biomaterials.

[16] Remya N.S., Syama S., Sabareeswaran A., Mohanan P.V. (2017). Investigation of chronic toxicity of hydroxyapatite nanoparticles administered orally for one year in wistar rats. Mater. Sci. Eng. C.

[17] Dan P., Sundararajan V., Ganeshkumar H., Gnanabarathi B., Subramanian A.K., Venkatasubu G.D., Ichihara S., Ichihara G., Sheik Mohideen S. (2019). Evaluation of hydroxyapatite nanoparticles—Induced in vivo toxicity in *Drosophila melanogaster*. Appl. Surf. Sci..

[18] Pappus S.A., Ekka B., Sahu S., Sabat D., Dash P., Mishra M. (2017). A toxicity assessment of hydroxyapatite nanoparticles on development and behaviour of *Drosophila melanogaster*. J. Nanoparticle Res..

[19] Zhao X., Ong K.J., Ede J.D., Stafford J.L., Ng K.W., Goss G.G., Loo S.C.J. (2013). Evaluating the Toxicity of Hydroxyapatite Nanoparticles in Catfish Cells and Zebrafish Embryos. Small.

[20] Geetha C.S., Remya N.S., Leji K.B., Syama S., Reshma S.C., Sreekanth P.J., Varma H.K., Mohanan P.V. (2013). Cells–nano interactions and molecular toxicity after delayed hypersensitivity, in Guinea pigs on exposure to hydroxyapatite nanoparticles. Colloids Surf. B Biointerfaces.

[21] Tampieri A., Landi E., Sandri M., Pressato D., Rivas Rey J., Bañobre-López M., Marcacci M. (2018). Intrinsically Magnetic Hydroxyapatite. (PCT/IB2011/053362). Patent No.

[22] Sandri M., Sprio S., Tampieri A. (2020). Physical solar filter consisting of substituted hydroxyapatite in an organic matrix. (WO2017/153888-102016000023614). Patent No.

[23] Adamiano A., Sangiorgi N., Sprio S., Ruffini A., Sandri M., Sanson A., Gras P., Grossin D., Francès C., Chatzipanagis K. (2017). Biomineralization of a titanium-modified hydroxyapatite semiconductor on conductive wool fibers. J. Mater. Chem. B.

[24] Tampieri A., Sandri M., Landi E., Pressato D., Francioli S., Quarto R., Martin I. (2008). Design of graded biomimetic osteochondral composite scaffolds. Biomaterials.

[25] Tampieri A., Iafisco M., Sandri M., Panseri S., Cunha C., Sprio S., Savini E., Uhlarz M., Herrmannsdörfer T. (2014). Magnetic Bioinspired Hybrid Nanostructured Collagen–Hydroxyapatite Scaffolds Supporting Cell Proliferation and Tuning Regenerative Process. ACS Appl. Mater. Interfaces.

[26] Panseri S., Cunha C., D’Alessandro T., Sandri M., Giavaresi G., Marcacci M., Hung C.T., Tampieri A. (2012). Intrinsically superparamagnetic Fe-hydroxyapatite nanoparticles positively influence osteoblast-like cell behaviour. J. Nanobiotechnol..

[27] OECD (2016). Test No. 220: Enchytraeid Reproduction Test, OECD Guidelines for the Testing of Chemicals.

[28] OECD (2012). Guidance on Sample Preparation and Dosimetry for the Safety Testing of Manufactured Nanomaterials.

[29] Ribeiro M.J., Maria V.L., Soares A.M.V.M., Scott-Fordsmand J.J., Amorim M.J.B. (2018). Fate and Effect of Nano Tungsten Carbide Cobalt (WCCo) in the Soil Environment: Observing a Nanoparticle Specific Toxicity in *Enchytraeus crypticus*. Environ. Sci. Technol..

[30] Schneider C.A., Rasband W.S., Eliceiri K.W. (2012). NIH Image to ImageJ: 25 years of image analysis. Nat. Methods.

[31] (2008). Soil Quality—Avoidance Test for Testing the Quality of Soils and Effects of Chemicals—Part 1: Test with Earthworms (*Eisenia fetida* and *Eisenia andrei*).

[32] Bicho R.C., Gomes S.I.L., Soares A.M.V.M., Amorim M.J.B. (2015). Non-avoidance behaviour in enchytraeids to boric acid is related to the GABAergic mechanism. Environ. Sci. Pollut. Res..

[33] Bicho R.C., Ribeiro T., Rodrigues N.P., Scott-Fordsmand J.J., Amorim M.J.B. (2016). Effects of Ag nanomaterials (NM300K) and Ag salt (AgNO_3_) can be discriminated in a full life cycle long term test with *Enchytraeus crypticus*. J. Hazard. Mater..

[34] Mendes L., Maria V., Scott-Fordsmand J., Amorim M. (2015). Ag Nanoparticles (Ag NM300K) in the Terrestrial Environment: Effects at Population and Cellular Level in *Folsomia candida* (Collembola). Int. J. Environ. Res. Public Health.

[35] Santos F.C.F., Gomes S.I.L., Scott-Fordsmand J.J., Amorim M.J.B. (2017). Hazard assessment of nickel nanoparticles in soil-The use of a full life cycle test with *Enchytraeus crypticus*. Environ. Toxicol. Chem..

[36] Guimarães B., Gomes S.I.L., Scott-Fordsmand J.J., Amorim M.J.B. (2022). Impacts of Longer-Term Exposure to AuNPs on Two Soil Ecotoxicological Model Species. Toxics.

[37] Li S., Jia M., Li Z., Ke X., Wu L., Christie P. (2021). Ecotoxicity of arsenic contamination toward the soil enchytraeid *Enchytraeus crypticus* at different biological levels: Laboratory studies. Ecotoxicol. Environ. Saf..

[38] Chen J., Dong X., Xin Y., Zhao M. (2011). Effects of titanium dioxide nano-particles on growth and some histological parameters of zebrafish (*Danio rerio*) after a long-term exposure. Aquat. Toxicol..

[39] He X., Reichl F.-X., Milz S., Michalke B., Wu X., Sprecher C.M., Yang Y., Gahlert M., Röhling S., Kniha H. (2020). Titanium and zirconium release from titanium- and zirconia implants in mini pig maxillae and their toxicity in vitro. Dent. Mater..

[40] Pereira C.M.S., Novais S.C., Soares A.M.V.M., Amorim M.J.B. (2013). Dimethoate affects cholinesterases in *Folsomia candida* and their locomotion—False negative results of an avoidance behaviour test. Sci. Total Environ..

[41] Landi E., Logroscino G., Proietti L., Tampieri A., Sandri M., Sprio S. (2008). Biomimetic Mg-substituted hydroxyapatite: From synthesis to in vivo behaviour. J. Mater. Sci. Mater. Med..

[42] Tampieri A., D’Alessandro T., Sandri M., Sprio S., Landi E., Bertinetti L., Panseri S., Pepponi G., Goettlicher J., Bañobre-López M. (2012). Intrinsic magnetism and hyperthermia in bioactive Fe-doped hydroxyapatite. Acta Biomater..

[43] Krishnakumar G.S., Gostynska N., Dapporto M., Campodoni E., Montesi M., Panseri S., Tampieri A., Kon E., Marcacci M., Sprio S. (2018). Evaluation of different crosslinking agents on hybrid biomimetic collagen-hydroxyapatite composites for regenerative medicine. Int. J. Biol. Macromol..

